# An atypical case of Takotsubo cardiomyopathy in a middle-aged male triggered by work-related stress: case report

**DOI:** 10.1093/ehjcr/ytag313

**Published:** 2026-05-05

**Authors:** Bassim Albizreh, Gagan Manoher, Fahmi Othman

**Affiliations:** Hamad Medical Corporation, Heart Hospital, Al-Rayan Road, PO Box 3050, Doha, Qatar; Hamad Medical Corporation, Heart Hospital, Al-Rayan Road, PO Box 3050, Doha, Qatar; Hamad Medical Corporation, Heart Hospital, Al-Rayan Road, PO Box 3050, Doha, Qatar

**Keywords:** Takotsubo cardiomyopathy, Stress-induced cardiomyopathy, Atypical presentation, Work-related stress, Transient left ventricular dysfunction, NSTEMI mimic, Normal coronary angiography, Case report

## Abstract

**Background:**

Takotsubo cardiomyopathy (TCM) is a reversible cardiomyopathy that mimics acute coronary syndrome and is most commonly observed in postmenopausal women following emotional stress. Presentation in middle-aged men is uncommon, and occupational stress is an increasingly recognized but underreported trigger.

**Case summary:**

A 55-year-old male with Type 2 diabetes mellitus, hypertension, and dyslipidaemia presented with prolonged mild central chest discomfort following an exceptionally long and stressful workday organizing a sporting event. High-sensitivity troponin T was elevated, and transthoracic echocardiography demonstrated reduced left ventricular ejection fraction with apical and mid-ventricular hypokinesia and basal sparing. Coronary angiography revealed no obstructive coronary artery disease. Repeat echocardiography showed near-complete recovery of left ventricular function within 3 days and complete normalization at 6-month follow-up.

**Discussion:**

This case illustrates an atypical presentation of TCM in a middle-aged male precipitated by prolonged occupational stress. It underscores the importance of considering TCM in patients presenting with acute coronary syndrome-like symptoms irrespective of sex and highlights work-related stress as a significant non-traditional trigger.

Learning points

- Takotsubo cardiomyopathy can mimic non-ST-elevation myocardial infarction/myocardial infarction with non-obstructive coronary arteries in men with cardiovascular risk factors, requiring coronary angiography for differentiation.
- Prolonged occupational stress with sleep deprivation is a recognized trigger for apical ballooning pattern on echocardiography.
- Rapid left ventricular function recovery after supportive therapy distinguishes Takotsubo from myocarditis or demand ischaemia.

## Introduction

Takotsubo cardiomyopathy (TCM) is an acute, reversible cardiomyopathy often mimicking acute coronary syndrome (ACS), characterized by transient regional systolic dysfunction with typical apical ballooning and basal sparing of the left ventricle.^1^ It predominantly affects postmenopausal women and is frequently triggered by emotional or physical stress.^2,3^ Although uncommon in males, recent reports suggest increasing recognition in younger patients and males, often with atypical triggers. This report presents an unusual case of TCM in a middle-aged male precipitated by work-related stress, aiming to broaden the understanding of TCM triggers and demographic spectrum.

## Summary figure

**Table:** 

Timepoint	Event/finding
02:00	End of 18-h work shift (sporting event)
02:30	Onset of central burning chest discomfort
12:30	Emergency department (ED) presentation; troponin T elevated (526 ng/L)
Day 1	Angiography (clear) and transthoracic echocardiography (TTE) [left ventricular ejection fraction (LVEF) 44%, apical ballooning]
Day 4	Repeat TTE: rapid recovery of systolic function
6 months	Follow-up: LVEF normal; asymptomatic

## Case history

A 55-year-old male with a 15-year history of Type 2 diabetes mellitus, hypertension, and dyslipidaemia presented to the ED with mild central chest discomfort described as a burning sensation. The symptom developed as he was going to sleep after an unusually long and stressful day spent moderating and organizing a sporting event, which concluded at ∼2:00 a.m. The discomfort persisted for ∼10 h, prompting his ED visit.

## Examination

On presentation, the patient was cooperative and not in distress. Physical examination showed normal vital signs, clear chest to auscultation, and normal heart sounds.

## Differential diagnosis/investigation/treatment

Initial high-sensitivity troponin T was elevated at 526 ng/L, with subsequent values of 434 and 424 ng/L. Electrocardiography (ECG) showed sinus rhythm, left axis deviation, and T-wave inversions in anterolateral leads (*Figure 1*), and the patient was admitted with a diagnosis of non-ST-elevation myocardial infarction (NSTEMI), and guideline-directed medical therapy was initiated, including dual antiplatelet therapy, anticoagulation, and beta-blocker. The patient was already on angiotensin converting enzyme inhibitor (ACEi) for his hypertension and was continued on that.

**Figure 1:**
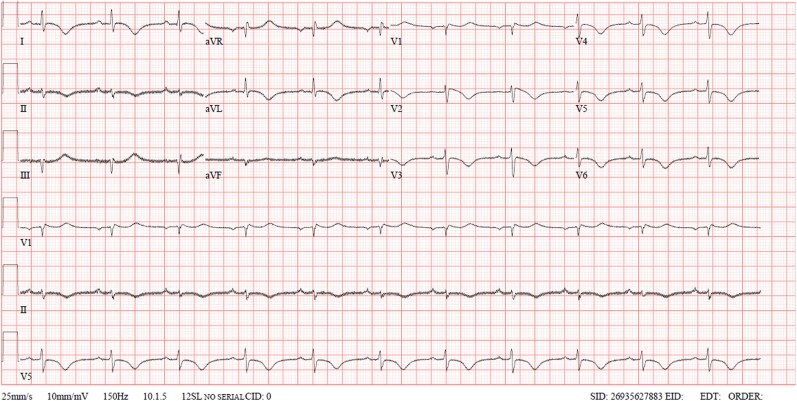
Admission electrocardiography showing normal sinus rhythm, left axis deviation, and T-wave inversions in anterolateral leads.

Transthoracic echocardiography on admission demonstrated a reduced LVEF of 44%, with diffuse hypokinesia of the apical and mid-ventricular segments, and relative sparing of the basal segments—consistent with apical ballooning (*Figure 2*).

**Figure 2:**
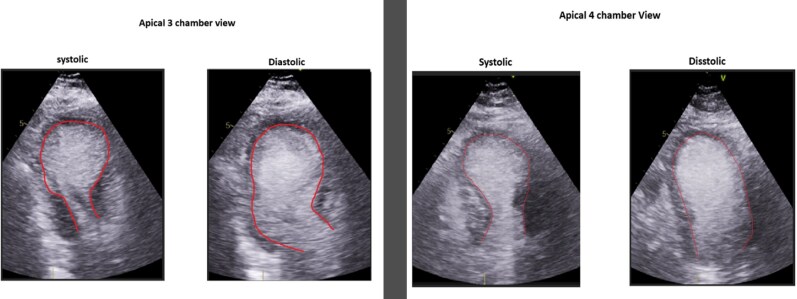
Admission transthoracic echocardiogram (apical four-chamber view) demonstrating reduced left ventricular ejection fraction with hypokinesia of the mid- and apical segments and relative basal sparing, compatible with apical ballooning.

The patient underwent urgent invasive coronary angiography, which revealed no evidence of obstructive coronary artery disease or plaque rupture (*Figure 3*), effectively ruling out an acute atherothrombotic event. Following angiography, differential diagnosis included myocardial infarction with non-obstructive coronary arteries (MINOCA), myocarditis, demand ischaemia, and TCM, which was strongly suspected given the extreme stressor, characteristic wall motion pattern on imaging, and the troponin rise disproportionate to the patient’s clinical stability. Myocarditis was considered less likely due to the lack of prodromal symptoms or fever, nor could demand ischaemia alone explain the focal wall motion abnormalities observed. Consequently, antiplatelet and anticoagulant therapy was discontinued, and the focus shifted to supportive heart failure management with a beta-blocker, sodium-glucose cotransporter 2 inhibitor (SGLT2i), and an ACE inhibitor.

**Figure 3:**
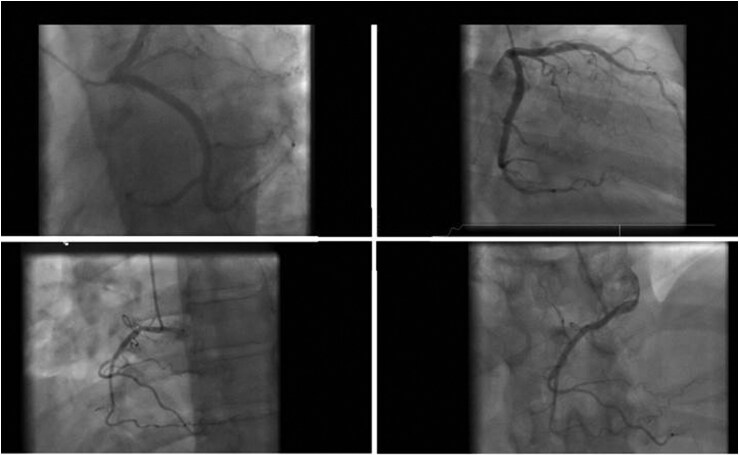
Coronary angiography showing unobstructed coronary arteries without evidence of culprit stenosis or plaque rupture.

## Outcome and follow-up

A repeat echocardiogram 3 days later showed near-complete normalization of LV function (EF ∼ normal range), with only mild residual hypokinesia at the apical cap (*Figure 4*). Follow-up TTE at 6 months revealed complete recovery of wall motion abnormalities and normal systolic function At that time, the SGLT2 inhibitor was discontinued following normalization of ventricular function on repeat imaging, whereas the beta-blocker and ACE inhibitor were continued beyond 6 months for hypertension management.

**Figure 4:**
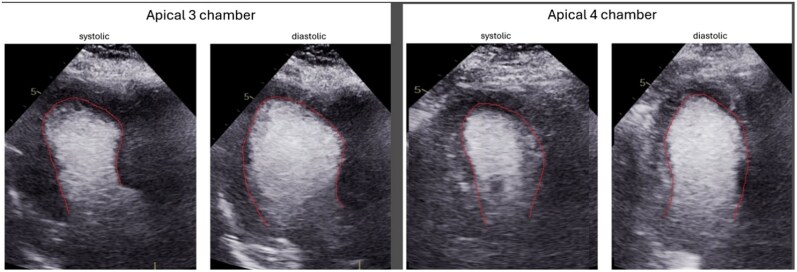
Follow-up echocardiogram (72 h) Follow-up transthoracic echocardiogram 3 days after presentation showing near-normalization of left ventricular systolic function with resolution of prior apical ballooning.

## Conclusion

Takotsubo cardiomyopathy should remain a differential diagnosis in patients presenting with ACS-like symptoms, even among middle-aged men with cardiovascular risk factors. Recognition of non-traditional stressors, such as prolonged occupational strain, is essential to prevent misdiagnosis and ensure appropriate management of this reversible condition.

## Discussion

This case meets the diagnostic criteria for TCM as per the International Takotsubo Diagnostic Criteria (InterTAK) including (i) transient regional wall motion abnormalities (apical ballooning) extending beyond a single epicardial vascular distribution, (ii) the presence of an emotional/physical stressor, (iii) new ECG abnormalities and elevated cardiac biomarkers, and (iv) the absence of obstructive coronary artery disease or evidence of acute plaque rupture on angiography. The complete recovery of ventricular function, confirmed on follow-up echocardiography, further solidifies the diagnosis.

The underlying pathophysiology is thought to be driven by a surge in catecholamines induced by the intense stressor. This surge can lead to direct myocardial stunning, microvascular dysfunction, and epicardial spasm, resulting in the characteristic pattern of wall motion abnormalities in the absence of obstructive coronary disease. The mid- and apical segments of the left ventricle are particularly vulnerable to these catecholamine-mediated effects due to a higher density of beta-adrenoceptors.

What makes this case particularly interesting is the patient’s demographic profile and the specific trigger for his condition. Takotsubo cardiomyopathy is most commonly observed in postmenopausal women, with men accounting for <10% of cases. Although TCM is typically triggered by intense emotional stress, such as grief, physical and occupational stressors are also increasingly recognized as potential contributors. In this case, the patient’s prolonged work-related stress likely caused a surge in catecholamines, leading to myocardial stunning and subsequent cardiac dysfunction.

The recognition of occupational strain as a primary catalyst for TCM is an evolving area of cardiovascular research. While traditional triggers often involve domestic bereavement, recent literature highlights that work-related stressors specifically those involving high stakes, long hours, and sleep deprivation can be equally cardiotoxic. Case reports have documented similar occurrences in high-pressure environments; for instance, cases have been reported in teachers following intense workplace conflict^4^ and in healthcare workers during the peak of the coronavirus disease 2019 (COVID-19) pandemic.^5^ These cases, like the one presented here, often share a common thread of exhaustion-induced vulnerability.

This case therefore supports the growing recognition of TCM in atypical populations and underscores the need to consider occupational or chronic stress as valid triggers, especially in males with underlying cardiovascular risk factors. It highlights the importance of eliciting a detailed occupational history in all patients presenting with acute cardiac syndromes.

## Key clinical message

Takotsubo cardiomyopathy, although more common in older women, can affect middle-aged men with cardiovascular risk factors. Physical or emotional work-related stress may trigger a presentation that mimics NSTEMI or MINOCA, with troponin elevation and regional wall motion abnormalities, often prompting invasive angiography. Recognition of the characteristic imaging pattern and absence of obstructive coronary disease is crucial, as TCM typically shows rapid and complete recovery of ventricular function with supportive therapy.

## Data Availability

The data that support the findings of this study are available in the supplementary material of this article.

## Lead author biography

Dr Bassim Albizreh is an associate consultant in cardiology at Heart Hospital, Doha, Qatar. He completed his Arab Board in Internal Medicine and Cardiology fellowship at Hamad Medical Corporation in Doha, Qatar. He has been a member of the Gulf Heart Association since 2015 and of the Royal College of Physicians of London since 2017. His clinical interests include cardiac imaging, acute coronary syndromes, and heart failure. He has several publications in peer-reviewed journals.

## Notes

**Consent:** The authors confirm that written consent for submission and publication of this case report including images and associated text has been obtained from the patient, in line with the COPE guidelines.
